# Reliability of a gamified tablet-based tapping digital test for assessing motor coordination in adults

**DOI:** 10.3389/fpubh.2025.1704140

**Published:** 2026-01-15

**Authors:** Yasmim Fernandes Moniz, Luis Duarte Andrade Ferreira

**Affiliations:** 1 NEUROREHABLAB, ARDITI - Agência Regional para o Desenvolvimento da Investigação, Tecnologia e Inovação, Funchal, Portugal; 2 Universidade da Madeira, Funchal, Portugal; 3 Universidade de Mogi das Cruzes, São Paulo, Brazil

**Keywords:** assessment, box and blocks, motor coordination, reliability, tablet, test

## Abstract

Motor coordination can be defined as the ability to perform motor tasks efficiently and in the shortest possible time. This skill is essential in daily life, as many tasks require a certain level of coordination. Assessing motor coordination is crucial, particularly in motor rehabilitation, making the availability of accessible and effective tools to measure this variable essential for clinical practice. The objective of this study was to verify the reliability of a new gamified motor coordination test (Tapping Digital Test on a tablet interface) and to investigate the correlation of this test with traditional assessment methods, including the Tapping Test, Box and Blocks Test, and Nine Hole Peg Test. A total of 45 healthy adults were recruited to participate, performing all motor coordination tests across two assessments with a 1-week interval between them. The results showed good intra-rater reliability (ICC of 0.80 for the right hand, *p* < 0.001, and 0.85 for the left hand, *p* <0.001). The Tapping Digital Test demonstrated moderate correlation with the Tapping Test (0.7 for the right hand and 0.42 for the left hand), while correlations with the other tests were weak. The gamified Tapping Digital Test stands out as a novel tool for assessing fine motor coordination, demonstrating good intra-rater reliability in healthy adults. Future studies should focus on establishing normative scores for the test and investigating its usability and reliability within clinical populations.

## 1 Introduction

Motor coordination can be defined as the ability to perform a motor task efficiently and in the shortest possible time (1, 2). This variable is very important in our daily lives, as many of the tasks we perform require a certain level of coordination. Good coordination requires full integration of the neuromuscular and sensorimotor systems (3).

The evaluation of motor coordination provides an indication of a person's motor functionality and is highly relevant in clinical practice, particularly in the health-disease process, diagnosis, and motor rehabilitation (4). To assess this variable, tests typically involve task-time situations, where the person must perform a standardized movement or task correctly in the shortest time possible. Examples of validated and widely used tests in clinical practice include the Box and Blocks Test, Nine Hole Peg Test, and Tapping Test (5–7).

However, precision and standardization in motor coordination assessment are crucial for obtaining reliable measurements, especially when the tests are used to evaluate progress in motor recovery. One solution to address this is the development of digital tools to measure motor coordination. The advantages of such systems include immediate time recording, greater accuracy, and reduced variability or error between different assessments (8–10). Digital evaluations of motor performance are increasingly becoming valuable tools in both research and clinical practice, primarily due to their ability to capture fine-grained temporal and spatial data that traditional tests often miss. For example, smartphone-based motor assessments offer low-cost, scalable solutions for monitoring motor function in underserved populations (11).

Similarly, wearable sensor- and game-based platforms have been successfully applied to quantify motor skills and coordination in neurological conditions, enabling objective, remote monitoring of changes in motor control (12).

The use of validated digital tools can facilitate and enhance the assessment of physiotherapy treatment progression. Such tools may be particularly beneficial for patients with Parkinson's disease and those undergoing post-stroke rehabilitation. Moreover, easily accessible digital measures can be seamlessly integrated into hybrid treatment approaches and e-health frameworks (13).

In a previous study, a new motor coordination test (9) (Available in: https://moniz-games.itch.io/tapping-digital-test-version2), the Tapping Digital Test (TDT), was developed, inspired by the traditional Tapping Test but implemented using a specific controller connected to a mobile device (9). Although the system was highly rated for usability, receiving the classification “Best Imaginable,” the initial investigation presented important methodological limitations. Test–retest reliability and temporal stability of performance were not assessed, which are fundamental elements to determine whether a digital instrument can be used in rehabilitation, longitudinal monitoring, or tele assessment. Furthermore, the weak correlations found between the original TDT and traditional motor coordination tests suggest that the instrument, in its original form, did not adequately reflect well-established motor constructs in the literature. The absence of analyses regarding potential relationships with physical fitness indicators also limits its applicability, as tapping tasks may reflect motor speed, rhythm, and resistance to repetitive effort, components relevant for functional assessments.

After a critical analysis of the system and the available results, it was identified that migrating to a tablet-based interface could offer substantial gains from both a technical and clinical perspective. Tablets allow greater precision in touch detection, wide commercial availability, higher standardization of the response area, and ease of integration into digital health environments. These characteristics enhance the potential of the TDT for use in physiotherapy, neurological rehabilitation, and remote monitoring, while also enabling integration with metrics related to physical fitness, such as motor speed and resistance to repetitive effort. Given these gaps and the clinical potential of a more accessible and technically robust instrument, the objectives of this study were: (1) to assess the reliability of the Tapping Digital Test (TDT) in its new tablet version; and (2) to investigate the correlation between this test and traditional motor coordination assessments, including the Tapping Test, Box and Blocks Test, and Nine Hole Peg Test, in order to establish initial evidence of validity for adults.

## 2 Methods

### 2.1 Study design

This research utilized a test-retest design. Participants completed all motor coordination tests, with two measurements collected 1 week apart. The study report follows the COSMIN (Connsensus-based Standards for the selection of health Measurement Instruments) reporting guidelines (14).

### 2.2 Participants

A total of 45 participants were recruited for this study to ensure robust estimation of test-retest reliability. This sample size is consistent with prior research in hand dexterity and upper limb motor function, where reliability studies have successfully obtained stable ICC estimates with similar or slightly smaller sample sizes. Recruiting 45 participants provides a conservative margin, enhancing the precision of the ICC estimates and minimizing the influence of measurement variability or outliers (15).

To participate in this research, individuals had to be between 18 and 60 years old. Participants with a history of upper limb injury in the last six months or any neuromuscular disease were excluded from the study. Only healthy individuals were included.

After obtaining consent to participate in the research, we collected the following information about the participants: age, gender, and dominant hand. A total of 45 volunteers were recruited from the University of Madeira (Portugal).

### 2.3 Tapping digital test

**Figure 1:**
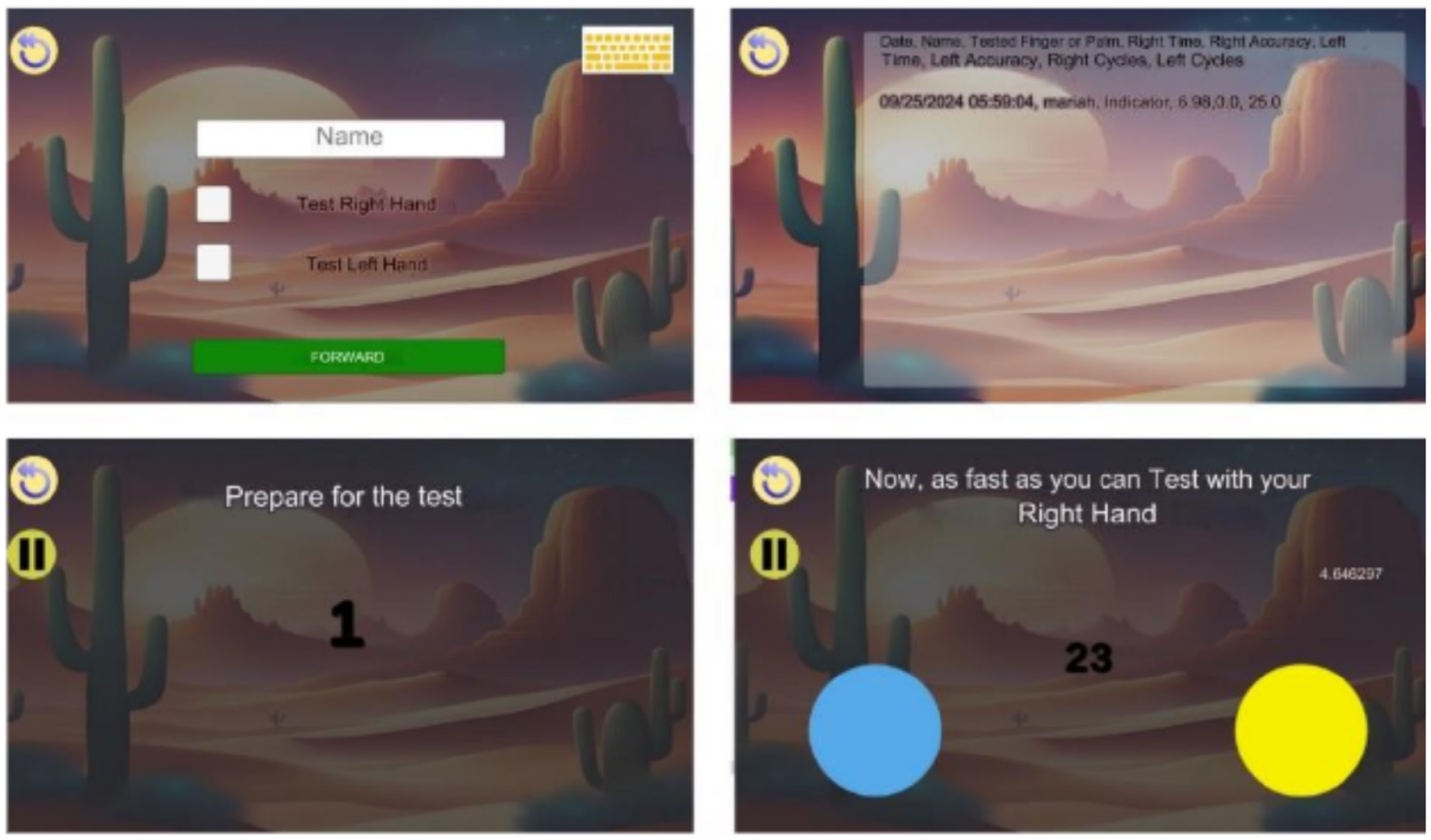
Interface tapping digital test.

The Tapping Digital Test was developed as an accessible instrument for measuring motor coordination on mobile devices, such as smartphones and tablets. The structure of this test was inspired by the traditional Tapping Test, where the tester alternately touches two circles as quickly as possible. The measurement is based on the time taken to complete 25 cycles (where touching one circle and then the other counts as one cycle). The system records the time taken for the tested hand. Once the test is completed, the data is automatically saved in the interface (Figure 1).

The game incorporated gamification principles into its overall design and conceptual framework. In the test, the primary objective was to present two visual targets, positioned, respectively, at the far right and far left of the screen. Participants were instructed to alternately touch each target with the same hand until completing a total of 25 cycles. Vibrant colors were employed to highlight the circular visual cues located at both extremities of the display. At the beginning of the test, an auditory signal indicated the commencement of the task, and throughout its execution, a real-time counter displayed the number of cycles completed. And the results are available in the application too.

The test can be conducted using a mobile application (for Android devices) or through a specific web page in a browser. The recommendations for the test follow the same protocols as standard motor coordination tests. Participants should be seated comfortably in a chair with support, positioned in front of a desk with the tablet placed at the center.

The examiner or tester selects the appropriate options for the test (including the tested hand and finger to be used), and the user is allowed to use only one finger during the test. The test begins once the countdown timer finishes.

### 2.4 Motor coordination tests

For the traditional Tapping Test, two circles of standard size are needed, along with a fixed rectangle on the table. The participant places their hand that will not be tested on the rectangle while the hand being evaluated touches one circle and then the other, tapping as quickly as possible. The evaluator announces the start and finish of the test and times the duration required to complete the task.

The Box and Blocks Test utilizes a wooden platform with 150 cubes and two compartments separated by a small wall. In this test, the participant must move the cubes one by one from one compartment to the other within a 60-s time limit. The number of cubes successfully transferred is recorded.

The Nine Hole Peg Test involves a plastic platform with one side designated for storing the pegs and the other side fitted vertically for inserting the pegs. The participant must take each peg one by one from the storage side and place it into the fitted section. Once all the pegs have been placed, the participant then removes them one by one and returns them to the original side. The measurement for this test is the total time taken to complete the task.

### 2.5 Procedures

Two measurements were taken for this study, with a 1-week interval between them. The procedures were conducted at the University of Madeira/Arditi (June–October of 2024) in an isolated room with a controlled environment, adhering to the standard protocols for assessing motor coordination as previously mentioned. Before each test, the examiner explained how the test works and provided a brief demonstration of what is expected and what is not acceptable in that specific test (for example, taking two cubes at a time in the Box and Blocks Test). After this short explanation, the examiner confirmed whether the volunteer was ready to begin, followed by a countdown to indicate when to start.

For the TDT, a 10.5-inch tablet running Android 13 was used, and volunteers were instructed to use only their index finger to standardize data collection. The two visual markers were circular, each with a diameter of 4cm, and the distance between their centers was 14cm.

All participants completed all motor coordination tests (TDT, Tapping Test, Box and Blocks Test, and Nine Hole Peg Test). The order of the tests was always the same: Tapping Test, TDT, Nine Hole Peg Test, and Box and Blocks Test. This sequence was adopted to minimize fatigue, starting with the tests that could be completed more quickly. A 30-s pause was provided between tests. Results were not disclosed to the participants; this information was held solely by the examiner. Instructions for the tests were provided in Portuguese, including those for the TDT. The examiner was a physiotherapist with experience in motor coordination assessment, and all evaluations were conducted by her.

After data collection, the correlation and reliability of the TDT were analyzed.

### 2.6 Data analyses

Descriptive statistics were utilized to summarize the population information. The Shapiro–Wilk test was conducted to assess the normality of the data, revealing that it followed a non-parametric distribution. To address the first objective, we calculated the intraclass correlation coefficients (ICC) using a two-way mixed model. The interpretation of the ICC values was as follows: less than 0.5 indicated poor reliability, between 0.5 and 0.75 indicated moderate reliability, between 0.75 and 0.9 indicated good reliability, and greater than 0.9 indicated excellent reliability [13].

Additionally, we assessed the agreement between the Tapping Test and the TDT using Bland-Altman analysis.

For our second objective, which focused on the correlation between the motor coordination tests, we performed Spearman correlation analysis, applying a significance level of <0.05 for each test. The interpretation of the correlation coefficients was as follows: values between 0.0 and 0.4 indicated weak correlation, between 0.5 and 0.8 indicated moderate correlation, and greater than 0.8 indicated strong correlation [10, 14]. All statistical analyses were conducted using SPSS software (version 29).

### 2.7 Ethical aspects

This research was conducted in accordance with the Declaration of Helsinki and received approval from the Ethics Committee of the University of Madeira (Portugal), under the number 109. Additionally, it was registered in Clinical Trials (NCT number: NCT06378411).

## 3 Results

A total of 45 volunteers participated in this study, all of whom completed the experimental protocol. Among the participants, 82% (37) were women, the ages ranging from 18 to 50 years old (average age: 27.53, SD ±6.68). Almost all participants were right-hand dominant (44).

### 3.1 Intra-rater reliability

Intra-rater reliability was assessed using the intraclass correlation coefficient (ICC), comparing Evaluation 1 with Evaluation 2 of the TDT. A two-way mixed consistency model was utilized. The ICC was calculated separately for the right and left hands. For the right hand, we obtained an ICC of 0.80 (95% CI: 0.60–0.93, *p* < 0.001), indicating good reliability and statistical significance. For the left hand, the ICC was 0.85 (95% CI: 0.74–0.92, *p* < 0.001), also considered to have good reliability and statistical significance.

### 3.2 Correlation between the motor coordination tests

The correlation analysis was conducted using the measures from the first evaluation of the study. The correlation between the traditional tests and the TDT on the right side revealed a moderate and positive correlation with the Tapping Test (0.70, *p* < 0.01). In contrast, the correlations with the Box and Blocks Test (–0.13, *p* > 0.05) and the Nine Hole Peg Test (–0.01, *p* > 0.05) were negative and weak (see Table 1).

**Table 1:** Correlation between tests for the right hand.

		**TDT**	**TT**	**BB**	**NH**
TDT	Correlation coefficient	1	0.702**	–0.136	–0.015
	Sig. (2-tailed)	–	<0.001	0.374	0.921
TT	Correlation coefficient	0.702**	1	–0.300*	–0.032
	Sig. (2-tailed)	<0.001	–	0.046	0.835
BB	Correlation coefficient	–0.136	–0.300*	1	–0.166
	Sig. (2-tailed)	0.374	0.046	–	0.276
NH	Correlation coefficient	–0.015	–0.032	–0.166	1
	Sig. (2-tailed)	0.921	0.835	0.276	–

TDT, Tapping Digital Test; TT, Tapping Test; BB, Box and Blocks Test; NH, Nine Hole Peg Test.

*Correlation is significant at the 0.05 level (2-tailed); **Correlation is significant at the 0.01 level (2-tailed).

For the correlation between the TDT and the other tests for the left hand, a moderate and positive correlation was found with the Tapping Test (0.42, *p* < 0.01). In contrast, the correlation with the Box and Blocks Test was weak and negative (–0.04, *p* >0.05), while the correlation with the Nine Hole Peg Test was weak and positive (0.28, *p* > 0.05) (see Table 2).

**Table 2:** Correlation between tests for the left hand.

		**TDT**	**TT**	**BB**	**NH**
TDT	Correlation coefficient	1	0.425**	–0.046	0.285
	Sig. (2-tailed)	–	0.004	0.765	0.058
TT	Correlation coefficient	0.425**	1	0.103	0.208
	Sig. (2-tailed)	0.004	–	0.501	0.171
BB	Correlation coefficient	–0.046	0.103	1	–0.509**
	Sig. (2-tailed)	0.765	0.501	–	<0.001
NH	Correlation coefficient	0.285	0.208	–0.509**	1
	Sig. (2-tailed)	0.058	0.171	<0.001	–

TDT, Tapping Digital Test; TT, Tapping Test; BB, Box and Blocks Test; NH, Nine Hole Peg Test.

*Correlation is significant at the 0.05 level (2-tailed); **Correlation is significant at the 0.01 level (2-tailed).

Among the traditional tests, only two statistically significant correlations were found. For the left hand, a moderate and negative correlation was observed between the Nine Hole Peg Test and the Box and Blocks Test (–0.50, *p* < 0.01). Additionally, for the right hand, there was a weak and negative correlation between the Tapping Test and the Box and Blocks Test (–0.30, *p* < 0.05).

**Figure 2:**
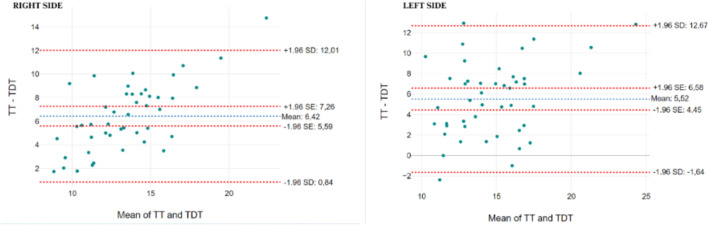
Bland-Altman plot of Tapping Test and Tapping Digital Test.

The Bland–Altman plot illustrates the agreement between the Tapping Digital Test (TDT) and the Tapping Test (see Figure 2). The mean difference between the Tapping Test and TDT by right side is 6.42, with limits of agreement ranging from 0.84 to 12.01 (±1.96 SD). Most data points fall within these limits, indicating good agreement between the two methods. A small bias is observed, with the Tapping Test consistently yielding higher values than the TDT. There is no clear trend of increasing or decreasing bias across the range of means, suggesting that the difference between the Tapping Test and TDT remains consistent as the values increase.

For the left side, the mean difference between the Tapping Test and TDT is 5.52, with limits of agreement ranging from –1.64 to 12.67 (±1.96 SD). The plot shows that the majority of points lie within the limits of agreement. The bias between the two tests is similar to that observed on the right side, with the Tapping Test generally producing slightly higher scores than the TDT, though no systematic bias is evident across the range of mean values.

## 4 Discussion

The intra-rater reliability of the TDT was deemed good for both hands, providing evidence that this digital measure can be effectively utilized as a valuable tool for assessing motor coordination. Reliability is a crucial parameter for health measurements as it ensures accuracy across different assessments. A strong intraclass correlation coefficient (ICC) indicates that the same rater will report consistent results when faced with the same scenario or measure during each evaluation. For clinical tests, it is essential that intra-rater reliability is at least considered good, anything less would be unacceptable for implementation in practice (16).

The high test-retest reliability observed for both hands is significant for clinical practice, as it allows for confident interpretation of results, even when assessing the non-dominant hand or the compromised side in populations with neurological conditions. This is a key differentiator of our study, as many validation studies typically focus only on the dominant hand. While this approach is understandable for tests specifically designed for unilateral assessment, it is crucial to investigate reliability for both sides in the context of a bilateral test. This ensures that the TDT is applicable and reliable across various clinical scenarios, enhancing its utility in evaluating motor coordination comprehensively (8, 17).

In previous study (9) was developed a controller designed for use with the TDT. However, we concluded that the controller, which featured five buttons, did not adequately replicate the concept of the traditional Tapping Test. As a result, we determined that using this controller would need to be considered as a completely different test. The correlation results between the Tapping Test and the TDT reflect this shift from the original concept. In the earlier study, did not find correlations exceeding weak levels, and none were statistically significant. The improved correlation in the current study indicates that the adjustments made have rendered the TDT more representative of the Tapping Test concept (13).

The correlation between the Tapping Test and the TDT was found to be moderate and positive, which was an expected outcome given that the Tapping Test served as the foundation for the development of this new test. The numerical differences observed between the right and left hands in the comparison of the two tests can be interpreted in light of the participants' handedness. Data for the dominant hand is likely to be more consistent. This is supported by the standard deviations reported in similar studies, which typically show that the non-dominant hand exhibits greater variability compared to the dominant hand. As a result, these findings highlight the importance of considering handedness when interpreting the results of motor coordination assessments (10, 15). However, in the case of the TDT, the correlation was defined as moderate for both hands, indicating that the test performs reliably regardless of laterality. This consistent level of correlation suggests that the TDT is effective in assessing motor coordination for both dominant and non-dominant hands, reinforcing its utility as a comprehensive evaluation tool in clinical settings (12).

The times (scores) of each test do not correspond directly. The Tapping Test tends to yield scores that are approximately 5–6 units higher than those of the TDT. This discrepancy can be justified by the greater amplitude required to perform the Tapping Test compared to the TDT, which necessitates more time to complete the task. Consequently, the inherent differences in task demands between the two tests likely account for the observed variations in timing (11).

In the other motor coordination tests, the correlations between the TDT and the Nine Hole Peg Test and Box and Blocks Test were found to be weak. This suggests that the TDT does not directly correspond with these traditional tests. This finding aligns with studies that have reported similarly weak correlations between the Nine Hole Peg Test and the Box and Blocks Test in patients with multiple sclerosis (6) and our previous study with 5 health people (9). Therefore, it can be concluded that the correlations between the various tests of motor coordination do not exhibit an equal distribution, and each test must be interpreted separately (18).

However, we confirmed two correlations: one between the Tapping Test and the Box and Blocks Test for the right side, and another between the Box and Blocks Test and the Tapping Test for the left side. These findings reveal a negative correlation, which is expected since the Box and Blocks Test measures performance in terms of the number of cubes moved rather than time.

Future studies could explore whether this data indicates a relationship between the tests or if it simply reflects a general trend in a person's motor coordination abilities, suggesting that proficiency in one test correlates with proficiency in another. In the case of a more direct relationship, such as that between the Tapping Test and the TDT, one would expect to see similar correlations for both hands.

### 4.1 Limitations and future studies

The study had several limitations. Our sample predominantly consisted of females and right-hand dominant individuals, which may affect the generalizability of the findings. Additionally, we did not assess interrater reliability; however, given the automated nature of the TDT, we hypothesize that future studies with this proposal will yield similar results regarding reliability. The consistent order in which the tests were performed might have had some impact on the measurements. Future studies could explore this hypothesis using a randomized design.

In this study, we used a 10-inch Samsung tablet (Galaxy A8) as it was the largest tablet available in our research lab. The test was validated using this specific configuration, and while it is possible to use other devices, variations in touchscreen size may affect the expected time to complete the task. Therefore, we recommend using the same device for comparisons. The app used on the tablet offers advantages, including stability, independence from internet connectivity (which likely reduces delays), and the ability to create a file with the tests conducted on that device.

Future studies should focus on establishing normative scores for the Tapping Digital Test in healthy populations and investigating its usability and reliability in clinical populations. This would enhance our understanding of the test's applicability across diverse groups and contexts.

### 4.2 Implications for practice

The Tapping Digital Test demonstrated good results for stability, indicating that this application, that can serve as an effective tool for evaluating fine motor coordination. It is a simple, completely free test that is accessible to both clinicians and patients, who can easily download the app or use it through an online browser.

Given the context of this application, its use may be highly valuable for telerehabilitation platforms, as it enables standardized assessment even when performed remotely, provided that the same evaluation conditions are maintained (including device type and positioning for repeated or progression measurements).

As the authors and developers of this test, we recommend adhering to a consistent protocol when administering the test to enable more accurate tracking of progress over time or in relation to therapeutic interventions.

However, it is important to note that the Tapping Digital Test does not replace traditional assessments such as the Nine Hole Peg Test and Box and Blocks Test. While the Tapping Digital Test shows a moderate correlation with the Tapping Test, it is essential to recognize that the Tapping Test generally yields longer times due to the larger amplitude of movement required for that task. Consequently, each test has its own specificities and should be interpreted in the context of its unique assessment parameters.

## 5 Conclusion

The Tapping Digital Test is a novel tool for assessing fine motor coordination, demonstrating good intra-rater reliability in healthy adults. The correlation between the Nine Hole Peg Test and Box and Blocks Test is weak, while the correlation with the Tapping Test is moderate for both hands. Future studies should focus on establishing normative scores for the Tapping Digital Test and further investigate its usability and reliability within clinical populations. This will help to enhance the understanding of its applicability in various contexts and patient groups.

## Data Availability

The raw data supporting the conclusions of this article will be made available by the authors, without undue reservation.
